# *trans*-Tetra­aqua­bis[3-(3-pyrid­yl)acrylato-κ*N*]cobalt(II)

**DOI:** 10.1107/S1600536808002559

**Published:** 2008-01-30

**Authors:** Jozef Miklovič, Jan Moncol, Dušan Mikloš, Peter Segľa, Marian Koman

**Affiliations:** a Department of Chemistry, Faculty of Natural Sciences, University of St. Cyril and Methodius, SK-91701 Trnava, Slovakia; b Department of Inorganic Chemistry, Slovak Technical University, Radlinského 9, SK-812 37 Bratislava, Slovakia

## Abstract

The asymmetric unit of the title compound, [Co(C_8_H_6_NO_2_)_2_(H_2_O)_4_], contains one half-mol­ecule. The Co^II^ atom lies on an inversion centre and is coordinated by two N atoms of the pyridine rings of 3-(3-pyrid­yl)acrylate anions and four O atoms of water mol­ecules in a distorted octa­hedral coordination geometry. In the crystal structure, inter­molecular O—H⋯O hydrogen bonds link the mol­ecules, forming a three-dimensional network.

## Related literature

For related literature, see: Ayyappan *et al.* (2001 ▶); Kurmoo *et al.* (2005 ▶); Tong *et al.* (2003 ▶); Zhou *et al.* (2006 ▶); For related structures, see: Huang *et al.* (2005 ▶); Tang *et al.* (2006 ▶); Yang *et al.* (2006 ▶).
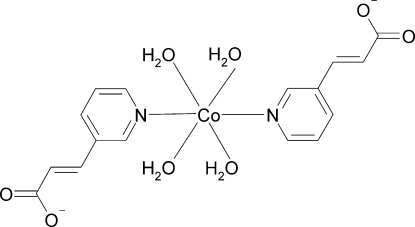


## Experimental

### 

#### Crystal data

[Co(C_8_H_6_NO_2_)_2_(H_2_O)_4_]
*M* *_r_* = 427.27
Monoclinic, 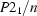

*a* = 11.235 (1) Å
*b* = 7.020 (1) Å
*c* = 12.012 (1) Å
β = 112.81 (1)°
*V* = 873.29 (18) Å^3^
*Z* = 2
Mo *K*α radiation
μ = 1.03 mm^−1^
*T* = 294 (2) K
0.40 × 0.25 × 0.20 mm

#### Data collection

Siemens *P*4 diffractometer
Absorption correction: ψ scan (*XEMP*; Siemens, 1994 ▶) *T*_min_ = 0.672, *T*_max_ = 0.808
3307 measured reflections
2533 independent reflections
2255 reflections with *I* > 2σ(*I*)
*R*_int_ = 0.030
3 standard reflections every 97 reflections intensity decay: 2.5%

#### Refinement

*R*[*F* ^2^ > 2σ(*F* ^2^)] = 0.038
*wR*(*F* ^2^) = 0.156
*S* = 1.10
2533 reflections
124 parameters
H-atom parameters constrained
Δρ_max_ = 0.65 e Å^−3^
Δρ_min_ = −0.84 e Å^−3^

### 

Data collection: *XSCANS* (Siemens, 1994 ▶); cell refinement: *XSCANS*; data reduction: *XSCANS*; program(s) used to solve structure: *SHELXS97* (Sheldrick, 2008 ▶); program(s) used to refine structure: *SHELXL97* (Sheldrick, 2008 ▶); molecular graphics: *ORTEP-3* (Farrugia, 1997 ▶); software used to prepare material for publication: *enCIFer* (Allen *et al.*, 2004 ▶).

## Supplementary Material

Crystal structure: contains datablocks global, I. DOI: 10.1107/S1600536808002559/hk2421sup1.cif

Structure factors: contains datablocks I. DOI: 10.1107/S1600536808002559/hk2421Isup2.hkl

Additional supplementary materials:  crystallographic information; 3D view; checkCIF report

## Figures and Tables

**Table:** 

Co—O1*W*	2.0895 (16)
Co—O2*W*	2.1061 (16)
Co—N1	2.1765 (18)

**Table:** 

O1*W*—Co—O2*W*	89.02 (6)
O1*W*—Co—N1	90.78 (7)
O2*W*—Co—N1	87.20 (7)

**Table 2:** Hydrogen-bond geometry (Å, °)

*D*—H⋯*A*	*D*—H	H⋯*A*	*D*⋯*A*	*D*—H⋯*A*
O2*W*—H3*W*⋯O2^i^	0.82	1.95	2.764 (3)	175
O2*W*—H4*W*⋯O2^ii^	0.82	1.95	2.741 (2)	161
O1*W*—H2*W*⋯O1^i^	0.82	1.86	2.678 (2)	175
O1*W*—H1*W*⋯O2^iii^	0.82	2.00	2.798 (2)	163

Symmetry codes: (i) 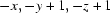; (ii) 
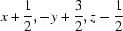
; (iii) 
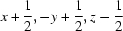
.

**Fig. 1.**
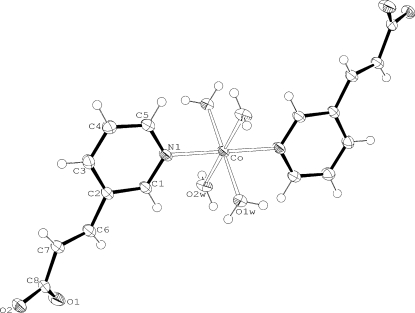
The molecular structure of the title molecule, with the atom-numbering scheme. Displacement ellipsoids are drawn at the 50% probability level

**Fig. 2.**
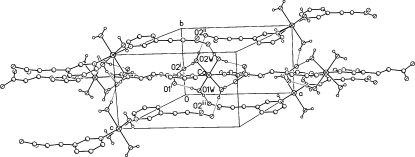
A packing diagram of (I). Hydrogen bonds are shown as dashed lines [symmetry codes: (i) -x, -y + 1, -z + 1; (ii) x + 1/2, -y + 3/2, z - 1/2; (iii) x + 1/2, -y + 1/2, z - 1/2].

## supplementary crystallographic information

### Comment

Several Co^II^ coordination polymers that contain a bridging 3-(3-pyridyl) -acrylate ligands have been reported recently (Ayyappan *et al.*, 2001; Kurmoo *et al.*, 2005; Tong *et al.*, 2003; Zhou *et al.*, 2006). However, if the 3-(3-pyridyl)-acrylate anions are coordinated only as terminal ligands, the possibility of participating in a hydrogen-bonding network originates. As part of our efforts to investigate metal(II) complexes based on pyridyl- carboxylic acids, we report herein the crystal structure of the title compound, (I).

In the molecule of the title compound, (I), (Fig. 1) Co^II^ atom lies on an inversion centre and adopts a distorted octahedral coordination geometry with two N atoms of the pyridine rings of 3-(3-pyridyl)-acrylate anions and four O atoms of water molecules (Table 1), where the two symmetry related 3-(3-pyridyl)-acrylate ligands are in *trans* positions.

The bond lengths and angles may be compared with the corresponding values in [Co(C_8_H_6_NO_2_)_2_(H_2_O)_4_].2H_2_O [(II); Huang *et al.*, 2005]. In (II), Co^II^ atom displays a similar distorted octahedral coordination geometry, but the existence of two more water molecules result in the formation of a different hydrogen-bonding network. On the other hand, complex (I) is isostructural with [Zn(C_8_H_6_NO_2_)_2_(H_2_O)_4_] [(III); Tang *et al.*, 2006; Yang *et al.*, 2006] and [Mn(C_8_H_6_NO_2_)_2_(H_2_O)_4_] [(IV); Huang *et al.*, 2005].

In the crystal structure, intermolecular O—H···O hydrogen bonds (Table 2) link the molecules to form a three-dimensional network.

### Experimental

Well shaped crystals of (I) suitable for X-ray analysis were prepared in an H-tube. In the first part of the H-tube, there was aqueous solutions of sodium salt of 3-(3-pyridyl)-acrylic acid and in the second part, there was aquous solution of Co(II) sulfate. The crystals formed after one month, they were separated and dried at room temperature (yield; 75%). The Anal. Calc.: C, 44.98; H, 6.61; N, 6.56; Co, 13.79; Found: C, 44.72; H, 6.31; N, 6.40; Co, 13.45. IR data (KBr) cm^-1^: 1646*m*ν(C?C); 1547vs ν_as_(COO^-^); 1371vs,br ν_s_(COO^-^); 649*m*δ(py); 415*m*χ(py). Electronic data (cm^-1^): 20200; 21200s h; 9100br.

### Refinement

H atoms were positioned geometrically with O—H = 0.82 Å (for OH_2_ and their displacement parameters were kept fixed as *U*_iso_ = 0.032 Å^2^) and C—H = 0.93 Å, for aromatic H atoms and constrained to ride on their parent atoms, with *U*_iso_(H) = 1.2*U*_eq_(C).

### Crystal data

**Table:**

[Co(C_8_H_6_NO_2_)_2_(H_2_O)_4_]	*F*_000_ = 442
*M**_r_* = 427.27	*D*_x_ = 1.625 Mg m^−^^3^
Monoclinic, *P*2_1_/*n*	Mo *K*α radiation λ = 0.71073 Å
Hall symbol: -P 2yn	Cell parameters from 25 reflections
*a* = 11.235 (1) Å	θ = 2.5–8.7º
*b* = 7.020 (1) Å	µ = 1.03 mm^−^^1^
*c* = 12.012 (1) Å	*T* = 294 (2) K
β = 112.81 (1)º	Block, pink
*V* = 873.29 (18) Å^3^	0.40 × 0.25 × 0.20 mm
*Z* = 2	

### Data collection

**Table:**

Siemens P4 diffractometer	*R*_int_ = 0.030
Radiation source: fine-focus sealed tube	θ_max_ = 30.0º
Monochromator: graphite	θ_min_ = 2.1º
*T* = 294(2) K	*h* = −1→15
2θ/ω scans	*k* = −1→9
Absorption correction: ψ scan(XEMP; Siemens, 1994)	*l* = −16→15
*T*_min_ = 0.672, *T*_max_ = 0.808	3 standard reflections
3307 measured reflections	every 97 reflections
2533 independent reflections	intensity decay: 2.5%
2255 reflections with *I* > 2σ(*I*)	

### Refinement

**Table:**

Refinement on *F*^2^	Secondary atom site location: difference Fourier map
Least-squares matrix: full	Hydrogen site location: inferred from neighbouring sites
*R*[*F*^2^ > 2σ(*F*^2^)] = 0.038	H-atom parameters constrained
*wR*(*F*^2^) = 0.156	*w* = 1/[σ^2^(*F*_o_^2^) + (0.1034*P*)^2^ + 0.5011*P*] where *P* = (*F*_o_^2^ + 2*F*_c_^2^)/3
*S* = 1.10	(Δ/σ)_max_ < 0.001
2533 reflections	Δρ_max_ = 0.65 e Å^−^^3^
124 parameters	Δρ_min_ = −0.84 e Å^−^^3^
Primary atom site location: structure-invariant direct methods	Extinction correction: none

### Special details

**Table:**

Geometry. All e.s.d.'s (except the e.s.d. in the dihedral angle between two l.s. planes) are estimated using the full covariance matrix. The cell e.s.d.'s are taken into account individually in the estimation of e.s.d.'s in distances, angles and torsion angles; correlations between e.s.d.'s in cell parameters are only used when they are defined by crystal symmetry. An approximate (isotropic) treatment of cell e.s.d.'s is used for estimating e.s.d.'s involving l.s. planes.
Refinement. Refinement of *F*^2^ against ALL reflections. The weighted *R*-factor *wR* and goodness of fit *S* are based on *F*^2^, conventional *R*-factors *R* are based on *F*, with *F* set to zero for negative *F*^2^. The threshold expression of *F*^2^ > σ(*F*^2^) is used only for calculating *R*-factors(gt) *etc*. and is not relevant to the choice of reflections for refinement. *R*-factors based on *F*^2^ are statistically about twice as large as those based on *F*, and *R*- factors based on ALL data will be even larger.

### Fractional atomic coordinates and isotropic or equivalent isotropic displacement parameters (Å^2^)

**Table:**

	*x*	*y*	*z*	*U*_iso_*/*U*_eq_	
Co	0.5000	0.5000	0.5000	0.01873 (16)	
N1	0.44388 (17)	0.5543 (3)	0.65137 (17)	0.0228 (4)	
O1	−0.13338 (15)	0.6559 (3)	0.61120 (16)	0.0328 (4)	
O2	−0.12936 (18)	0.5143 (2)	0.77834 (18)	0.0264 (4)	
O1W	0.38329 (15)	0.2573 (2)	0.45303 (14)	0.0257 (3)	
H1W	0.3959	0.1739	0.4111	0.032*	
H2W	0.3072	0.2884	0.4306	0.032*	
O2W	0.33927 (15)	0.6646 (2)	0.39394 (15)	0.0270 (3)	
H3W	0.2800	0.6092	0.3407	0.032*	
H4W	0.3567	0.7701	0.3751	0.032*	
C1	0.3185 (2)	0.5672 (3)	0.63318 (19)	0.0236 (4)	
H1	0.2578	0.5545	0.5546	0.028*	
C2	0.27363 (19)	0.5986 (3)	0.72477 (19)	0.0217 (4)	
C3	0.3653 (2)	0.6228 (3)	0.84170 (19)	0.0248 (4)	
H3	0.3400	0.6463	0.9055	0.030*	
C4	0.4946 (2)	0.6114 (4)	0.86108 (19)	0.0273 (4)	
H4	0.5574	0.6271	0.9384	0.033*	
C5	0.5303 (2)	0.5765 (3)	0.7649 (2)	0.0243 (4)	
H5	0.6177	0.5681	0.7795	0.029*	
C6	0.1337 (2)	0.6061 (3)	0.69088 (19)	0.0238 (4)	
H6	0.0838	0.6301	0.6100	0.029*	
C7	0.0699 (2)	0.5824 (3)	0.7628 (2)	0.0249 (4)	
H7	0.1153	0.5635	0.8452	0.030*	
C8	−0.07372 (19)	0.5859 (3)	0.71227 (19)	0.0216 (4)	

### Atomic displacement parameters (Å^2^)

**Table:**

	*U*^11^	*U*^22^	*U*^33^	*U*^12^	*U*^13^	*U*^23^
Co	0.0127 (2)	0.0260 (2)	0.0190 (2)	−0.00015 (12)	0.00773 (16)	−0.00068 (12)
N1	0.0157 (8)	0.0320 (9)	0.0231 (8)	−0.0009 (7)	0.0103 (6)	−0.0001 (7)
O1	0.0190 (7)	0.0495 (10)	0.0296 (8)	−0.0006 (7)	0.0091 (6)	0.0048 (7)
O2	0.0215 (8)	0.0310 (9)	0.0317 (9)	−0.0023 (6)	0.0158 (7)	−0.0007 (6)
O1W	0.0187 (7)	0.0311 (8)	0.0281 (8)	−0.0023 (6)	0.0098 (6)	−0.0046 (6)
O2W	0.0178 (7)	0.0323 (8)	0.0292 (8)	−0.0012 (6)	0.0072 (6)	0.0063 (6)
C1	0.0155 (8)	0.0353 (11)	0.0219 (9)	−0.0015 (8)	0.0092 (7)	−0.0026 (8)
C2	0.0167 (8)	0.0272 (9)	0.0240 (9)	−0.0002 (7)	0.0108 (7)	−0.0010 (7)
C3	0.0220 (9)	0.0341 (11)	0.0209 (9)	0.0016 (8)	0.0111 (8)	−0.0010 (8)
C4	0.0205 (9)	0.0390 (11)	0.0204 (9)	0.0006 (8)	0.0058 (7)	−0.0017 (8)
C5	0.0166 (9)	0.0301 (11)	0.0265 (10)	−0.0004 (8)	0.0086 (8)	−0.0012 (8)
C6	0.0168 (8)	0.0306 (10)	0.0252 (9)	0.0005 (7)	0.0097 (7)	−0.0011 (8)
C7	0.0169 (9)	0.0337 (11)	0.0256 (9)	−0.0012 (8)	0.0098 (7)	−0.0030 (8)
C8	0.0179 (8)	0.0248 (9)	0.0252 (9)	−0.0014 (7)	0.0117 (7)	−0.0046 (7)

### Geometric parameters (Å, °)

**Table:**

Co—O1W^i^	2.0895 (16)	C1—C2	1.394 (3)
Co—O1W	2.0895 (16)	C1—H1	0.9300
Co—O2W^i^	2.1061 (16)	C2—C3	1.393 (3)
Co—O2W	2.1061 (16)	C2—C6	1.465 (3)
Co—N1^i^	2.1765 (18)	C3—C4	1.382 (3)
Co—N1	2.1765 (18)	C3—H3	0.9300
N1—C5	1.342 (3)	C4—C5	1.384 (3)
N1—C1	1.343 (3)	C4—H4	0.9300
O1—C8	1.238 (3)	C5—H5	0.9300
O2—C8	1.288 (3)	C6—C7	1.329 (3)
O1W—H1W	0.82	C6—H6	0.9300
O1W—H2W	0.82	C7—C8	1.488 (3)
O2W—H3W	0.82	C7—H7	0.9300
O2W—H4W	0.82		
			
O1W^i^—Co—O1W	180.0	N1—C1—C2	124.1 (2)
O1W^i^—Co—O2W^i^	89.02 (6)	N1—C1—H1	118.0
O1W—Co—O2W^i^	90.98 (6)	C2—C1—H1	118.0
O1W^i^—Co—O2W	90.98 (6)	C3—C2—C1	117.57 (19)
O1W—Co—O2W	89.02 (6)	C3—C2—C6	124.77 (18)
O2W^i^—Co—O2W	180.0	C1—C2—C6	117.65 (19)
O1W^i^—Co—N1^i^	90.78 (7)	C4—C3—C2	118.75 (19)
O1W—Co—N1^i^	89.22 (7)	C4—C3—H3	120.6
O2W^i^—Co—N1^i^	87.20 (7)	C2—C3—H3	120.6
O2W—Co—N1^i^	92.80 (7)	C3—C4—C5	119.7 (2)
O1W^i^—Co—N1	89.22 (7)	C3—C4—H4	120.1
O1W—Co—N1	90.78 (7)	C5—C4—H4	120.1
O2W^i^—Co—N1	92.80 (7)	N1—C5—C4	122.64 (19)
O2W—Co—N1	87.20 (7)	N1—C5—H5	118.7
N1^i^—Co—N1	180.0	C4—C5—H5	118.7
C5—N1—C1	117.26 (18)	C7—C6—C2	127.3 (2)
C5—N1—Co	122.64 (14)	C7—C6—H6	116.4
C1—N1—Co	120.10 (14)	C2—C6—H6	116.4
Co—O1W—H1W	120.6	C6—C7—C8	120.3 (2)
Co—O1W—H2W	109.7	C6—C7—H7	119.8
H1W—O1W—H2W	113.2	C8—C7—H7	119.8
Co—O2W—H3W	117.2	O1—C8—O2	123.5 (2)
Co—O2W—H4W	114.9	O1—C8—C7	119.73 (19)
H3W—O2W—H4W	115.0	O2—C8—C7	116.8 (2)
			
O1W^i^—Co—N1—C5	−54.14 (19)	C1—C2—C3—C4	−1.1 (3)
O1W—Co—N1—C5	125.86 (19)	C6—C2—C3—C4	179.7 (2)
O2W^i^—Co—N1—C5	34.84 (19)	C2—C3—C4—C5	0.0 (4)
O2W—Co—N1—C5	−145.16 (19)	C1—N1—C5—C4	0.0 (4)
O1W^i^—Co—N1—C1	125.68 (19)	Co—N1—C5—C4	179.86 (18)
O1W—Co—N1—C1	−54.32 (19)	C3—C4—C5—N1	0.5 (4)
O2W^i^—Co—N1—C1	−145.34 (19)	C3—C2—C6—C7	−20.7 (4)
O2W—Co—N1—C1	34.66 (19)	C1—C2—C6—C7	160.2 (2)
C5—N1—C1—C2	−1.2 (4)	C2—C6—C7—C8	−177.2 (2)
Co—N1—C1—C2	178.93 (18)	C6—C7—C8—O1	−17.7 (3)
N1—C1—C2—C3	1.8 (4)	C6—C7—C8—O2	162.4 (2)
N1—C1—C2—C6	−179.0 (2)		

Symmetry codes: (i) −*x*+1, −*y*+1, −*z*+1.

### Hydrogen-bond geometry (Å, °)

**Table:**

*D*—H···*A*	*D*—H	H···*A*	*D*···*A*	*D*—H···*A*
O2W—H3W···O2^ii^	0.82	1.95	2.764 (3)	175
O2W—H4W···O2^iii^	0.82	1.95	2.741 (2)	161
O1W—H2W···O1^ii^	0.82	1.86	2.678 (2)	175
O1W—H1W···O2^iv^	0.82	2.00	2.798 (2)	163

Symmetry codes: (ii) −*x*, −*y*+1, −*z*+1; (iii) *x*+1/2, −*y*+3/2, *z*−1/2; (iv) *x*+1/2, −*y*+1/2, *z*−1/2.
